# How will our understanding of human development evolve over the next 10 years

**DOI:** 10.1038/s41467-021-24794-2

**Published:** 2021-07-29

**Authors:** Ali H. Brivanlou, Nicolas Rivron, Norbert Gleicher

**Affiliations:** 1 Laboratory of Synthetic Embryology The Rockefeller University, New York, NY USA; 2 Laboratory for Synthetic Development Institute of Molecular Biotechnology of the Austrian Academy of Sciences, Vienna Biocenter, Vienna, Austria; 3 The Center for Human Reproduction, New York, NY USA; 4 The Foundation for Reproductive Medicine, New York, NY USA; 5 Department of Obstetrics and Gynecology, Medical University of Vienna, Vienna, Austria

**Keywords:** Developmental biology, Embryogenesis

## Abstract

In the next 10 years, the continued exploration of human embryology holds promise to revolutionize regenerative and reproductive medicine with important societal consequences. In this Comment we speculate on the evolution of recent advances made and describe emerging technologies for basic research, their potential clinical applications, and, importantly, the ethical frameworks in which they must be considered.

## Future milestones in basic science of human development

In its infancy only a few years ago, the basic understanding of human development will continue its progress. The future holds significant promise for transformative discoveries about the origin of humans, driven by the development of new tools that synergistically combine biology with principles of physics, engineering and artificial intelligence (AI), within an appropriate ethical framework.

We can expect the deciphering of the complete molecular signatures of every human cell forming the *conceptus* (until gastrulation time^1^ and during fetal stages), allowing for the discovery of additional human-specific genetic features, molecular networks, cells and tissue types. This information will unveil human-specific functions, even in transient organs and tissues, such as our fetal gill, tail, or the subplate of our primitive brain^2^, which in turn will highlight evolutionary differences with classical model systems.

Ethical discussions regarding the utilization of genome editing technologies will, unquestionably, be at centerstage, due to their potential to illuminate the functions of (epi)genetics in development and diseases. How central this subject is to scientific progress, not only in reproductive biology and medicine, is well demonstrated by several recent reports including the conjoint one from the U.S. National Academy of Medicine and the Royal Society of the U.K., titled “*Heritable Human Genome Editing*.”^3^ These reports establish an initial framework for governments and other regulatory bodies worldwide to evaluate research involving human germline editing. In the next decade, this research will remain within the in vitro realm (e.g., human gametes or embryos cultured in vitro). Movement of germline editing into clinical practice can and should only occur after the steps outlined in the reports cited above have taken place. Therefore, it is currently difficult to imagine that germ line gene editing will be ethically accepted and technically ready for clinical practice within the next 10 years.

However, assessing the safety and efficacy of numerous biomedical approaches (e.g., to improve in vitro fertilization (IVF), contraception, and understand monogenic diseases) will require the ability to culture human embryos for a few weeks in vitro. We thus predict that in the next decade human embryos will likely be allowed to be maintained in vitro beyond the current 14-day limit according to guidelines generated by the appropriate oversight committee, as benefits linked to knowledge gained and potential clinical applications from such a step will outweigh the societal and cultural concerns that can still be held.

As an ethical alternative to the use of human and animal embryos, the emerging field of embryo modeling will form, through the self-organization of stem cells, increasingly sophisticated in vitro substitutes. Within the next decade, embryo models that do not attempt to recapitulate the development of the entire *conceptus* (e.g., *gastruloids*) will progress toward the first trimester, thus providing, for the first time, a blueprint of our early developmental origins (e.g., body axis formation, somitogenesis). In parallel, embryo models mimicking the entire *conceptus* (e.g., blastoids) will be combined, as in vitro discovery platforms, with the uterus organoid to expose the hidden processes of implantation and development that occur within the first weeks post fertilization. Although it is not conceivable to use human blastoids in reproduction in the coming ten years^4^, such in vitro platforms will guide drug and biomedical discoveries to better manage early pregnancy. Altogether, by recapitulating events that are otherwise impossible to access, embryo models will provide an ethical alternative toward addressing global health issues of family planning (e.g., high early pregnancy loss, contraception), developmental diseases (e.g., monogenic), and of their prevention according to the developmental origins of health and diseases.

Our basic knowledge of human organ generation and regeneration will also evolve. The combinations of primordial organoids through bottom-up approaches^5^ supported by engineered environments (e.g., hydrogels, microfluidics)^6^ and vasculatures^7^ will generate more standardized, mature and functional organs of increased volumes and complexities. Achieving the in vitro maturation of such complex organ systems using autologous human induced pluripotent stem cells (iPSCs) will open possibilities for more predictive and personalized drug-screens, and in the long-term, for therapeutic transplants. These systems will also shed light on currently inaccessible fundamental knowledge about human organogenesis and physiology, including new ontologies, which, through careful comparisons to species endowed with enhanced regenerative capacities (e.g., axolotl^8^) will reveal therapeutic targets to unleash potential human regenerative capacities, thus paving the way toward regenerative medicine’s long-term goals in humans.

## A new horizon for reproductive medicine induced by basic research

We foresee progress for the first clinical applications of burgeoning technologies that emerged over the last decade and for the development of clinical protocols that will revolutionize science and society.

In reproductive medicine one can foresee successful in vitro culture of primordial follicles toward maturity. Currently, only one out of 400 follicles will mature to give rise to a fertilizable oocyte, while the rest undergoes degeneration and apoptosis before reaching ovulation. Since this is a highly wasteful process, an improved in vitro maturation process would radically change current infertility treatments. Even at young ages, single egg retrieval currently only yields on average between 10 and 15 oocytes. A single small cortical ovarian biopsy at relatively young ages, in contrast, could offer a woman a potential egg pool of hundreds of oocytes. Improvement of in vitro primordial follicle culture could therefore virtually secure future conception for women at almost all ages. Women will then be afforded the same privilege as males, who, because of continuous spermatogenesis, are assured of genetic paternity into advanced age. Such a development would, of course, relieve women of considerable socio-biological pressures, while dramatically increasing gender equality. Studies to reach this goal are already underway^9^.

Further improvement of currently still sub-optimum cryopreservation methods of ovarian tissue, embryos and oocytes will provide security for women about to undergo often destructive cancer therapies or provide women of older ages the chance to have children later in life. In science, the resolution of one question, only leads to one or more new ones. Here, the most obvious arising question that will have to be answered is, up to which age should motherhood then be pursued in the view of child welfare?

Currently increasingly recognized clinical limitations of preimplantation genetic testing for aneuploidy (PGT-A)^10,11^, also addressed in our 10-year retrospective Comment, do not mean that novel cellular and molecular diagnostic approaches for embryo selection in IVF may not succeed in providing a more precise ranking methodology to evaluate embryo quality and live birth chances. As deep neural networks and AI, integrated with transcriptomic signatures, are increasingly introduced to IVF practice, progress can be expected^12^.

Another theme entering the field is the influences of the environment on the reproductive ecosystem. Notably, the role of the microbiome, which has been attracting increasing attention throughout medicine, and will, undoubtedly, also receive more prominent attention in reproductive sciences^13^. Additionally, the cellular and molecular mechanism underlying immune-tolerance pathways that are induced by the immune system in order to tolerate implantation of and invasion by a semi-allogeneic paternal graft will be, finally, elucidated^14^. Given the high prevalence of mosaicism, chromosomal instability in human preimplantation-stage embryos, it is tempting to hypothesize that what represents a detrimental and unlimited process in malignancies, may be a beneficial, yet only temporary, process in embryo implantation. Therefore, establishing what stops invasiveness in early pregnancy (except in cases of choriocarcinoma) may, therefore, have relevance for both reproductive and cancer biology.

To model reproductive diseases in a dish, stem cell-derived ovaries and testes will be modified using CRIPR/Cas9 genome editing tools in a manner similar to standardized organoids, described in our accompanying Comment^15^. These structures will be used in highly quantitative platforms to conduct high-throughput screens and identify drug candidates that can rescue any reproductive aberrance. When complemented with in vitro studies of embryo models (e.g., blastoids^16–18^) such in vitro investigations will lead to the development of drug treatments that will rescue early pregnancy defects, including failure to implant, affecting more than 40% of in vivo implanting blastocysts.

Ultimately, female and male independence from the age-effects on our own gonadal function would be further enhanced through the ability to produce gametes in vitro. This production would be achieved by reprogramming somatic cells into iPSCs and differentiating them into gametes. In the next decade, such a process is likely to radically change patient perspectives about their fertility potential into advancing age, once they can be given the ability to produce autologous sperm or eggs at all ages in vitro. This gametic production would also revolutionize how fertility services are currently offered to infertile patients and would allow same-sex couples conception with shared genetic parenthood^19^. In the mouse, male and female gametes have already been successfully produced from stem cells^20^, and multiple laboratories around the world are currently trying to repeat this accomplishment in humans.

## Ethical challenges

We believe that human embryology research and its ethical challenges will be increasingly seen as a driving force in addressing societal changes toward gender equality, and reduction in experimental use of animals and embryos. By establishing a more prominent dialogue between scientists, ethicists, funding/governmental agencies, and the public, we can build the medicine of tomorrow, including currently still largely unimaginable progress in fertility and pregnancy management, disease prevention and regenerative medicine. As previously noted, the most appropriate bodies addressing issues in reproductive research and clinical care are constantly challenged by scientific progress and adapting appropriate ethical frameworks to oversee the rapidly advancing field^3^.

As clinical perspectives become clear, ethical debates surrounding human embryology are likely to become centered around utilitarian arguments relative to health and diseases, rather than on cultural arguments, that are inherently diverse worldwide. Nevertheless, it is possible to foresee contradictory views on altering the genetic make-up of embryos, which would bring long-lasting genetic changes to future generations. The question will probably focus on whether there are clinical cases for which it would be ethical to modify preimplantation embryos considered as abnormal, with the intent of preventing pregnancy loss or diseases after birth. Whether such embryo transfers will become ethically acceptable is raised by two distinct clinical scenarios: the transfer of genetically-edited IVF embryos and the transfer of embryos produced by non-traditional means (e.g., issued from the fusion of gametes generated from genetically-edited stem cells). A recent opinion from the Ethics Committee of the ASRM under the heading *“Ethics in embryo research*” concluded that such research with reproductive intent should only be undertaken after pre-clinical research demonstrates acceptable levels of safety and efficacy and with the intent of improving the health and/or well-being of offspring^21^. Although this opinion does not offer a precise framework, it points at the necessity to develop in vitro tests (e.g., human embryos cultures, embryo models) to assess the safety/efficacy of potential therapeutic approaches to treat, for example, monogenic inherited diseases.

Ethical considerations will always determine the pace of scientific, medical, and societal progress and we believe that these considerations should be based on a holistic evaluation of the risks and benefits. As such, it is important to remember that science is driven by an international community and must thus respect all ethical frameworks when establishing regulatory guidelines. We remain confident that the advances in human embryology over the next ten years will provide a tremendous window into our own origins and contribute to positive medical and societal changes.
